# Aquitardifer: A New Hydrogeologic Term for Geologic Materials with both Aquitard and Aquifer Properties

**DOI:** 10.1111/gwat.70022

**Published:** 2025-09-08

**Authors:** Anthony C. Runkel, Jessica R. Meyer

**Affiliations:** ^1^ Department of Earth and Environmental Sciences University of Iowa Iowa City IA 52242

## Abstract

We propose that a new term, aquitardifer, be added to the hydrogeologic nomenclature. Aquitardifer, a blend of the terms aquitard and aquifer, accounts for geologic materials that have properties of both as traditionally defined. Several examples of aquitardifers are provided, as is justification for and applicability of the term.

## Introduction

An aquifer system traditionally is divided into multiple hydrogeologic units classified as either aquitards or aquifers based on their bulk hydraulic conductivity. With advances in hydrogeologic techniques such as borehole geophysics and multi‐level monitoring systems, there is increasing recognition that even relatively thin geologic units are commonly hydrogeologically complex in a manner such that they cannot be satisfactorily classified only as an aquifer or aquitard (nor related terms such as aquiclude or confining unit) using conventional definitions of those terms. As noted by Runkel et al. (2003) the definitions of aquifer and aquitard are not mutually exclusive. Recently published conventional definitions of each are provided by Sharp Jr. (2023): An aquifer is a saturated (consolidated or unconsolidated) geologic unit (material, stratum, or formation) or set of connected units that yield water of suitable quality to wells or springs in economically usable amounts; whereas an aquitard is a geologic material, stratum, or formation of low permeability (a confining unit) that transmits significant amounts of water only at a regional scale or over geologic time. An overriding problem is anisotropy to the degree whereby a geologic unit can yield economic quantities of water because of high hydraulic conductivity in one dimension and in the same location be of sufficiently low hydraulic conductivity in another dimension to act as an aquitard (Peffer 1991; Eaton and Bradbury 2003; Cherry et al. 2006; Eaton et al. 2007; Anderson et al. 2015; Runkel et al. 2018; Meyer et al. 2023).

The term aquitardifer, derived from blending parts of the words aquitard and aquifer, was first introduced by Runkel (2010) to account for geologic units in the Cambrian‐Ordovician aquifer system (COAS) of the North American midcontinent that have properties consistent with the conventional definitions of both aquifer and aquitard. Working in the same region, Anderson et al. (2011) applied the term locally to an Ordovician carbonate formation, and Runkel et al. (2018) suggested that a highly anisotropic siliciclastic interval of Cambrian strata is best classified as an aquitardifer at a regional scale. Extensive characterization of a DNAPL site in Wisconsin (Meyer et al. 2008, 2016, 2023), also in the COAS, likewise documented highly anisotropic aquitards, with horizontal hydraulic conductivity (Kh) values commonly similar to or even greater than the Kh values for the aquifers. These authors did not employ the term aquitardifer, but noted their results indicated that traditional aquitard and aquifer delineation was not sufficient to capture all the hydraulic contrasts important to flow and contaminant transport (Meyer et al. 2008). Beyond these bedrock examples, heterogeneous unconsolidated materials with high anisotropy, such as some of those compiled by Shepley (2024), also could best be classified as aquitardifers.

**Figure 1:**
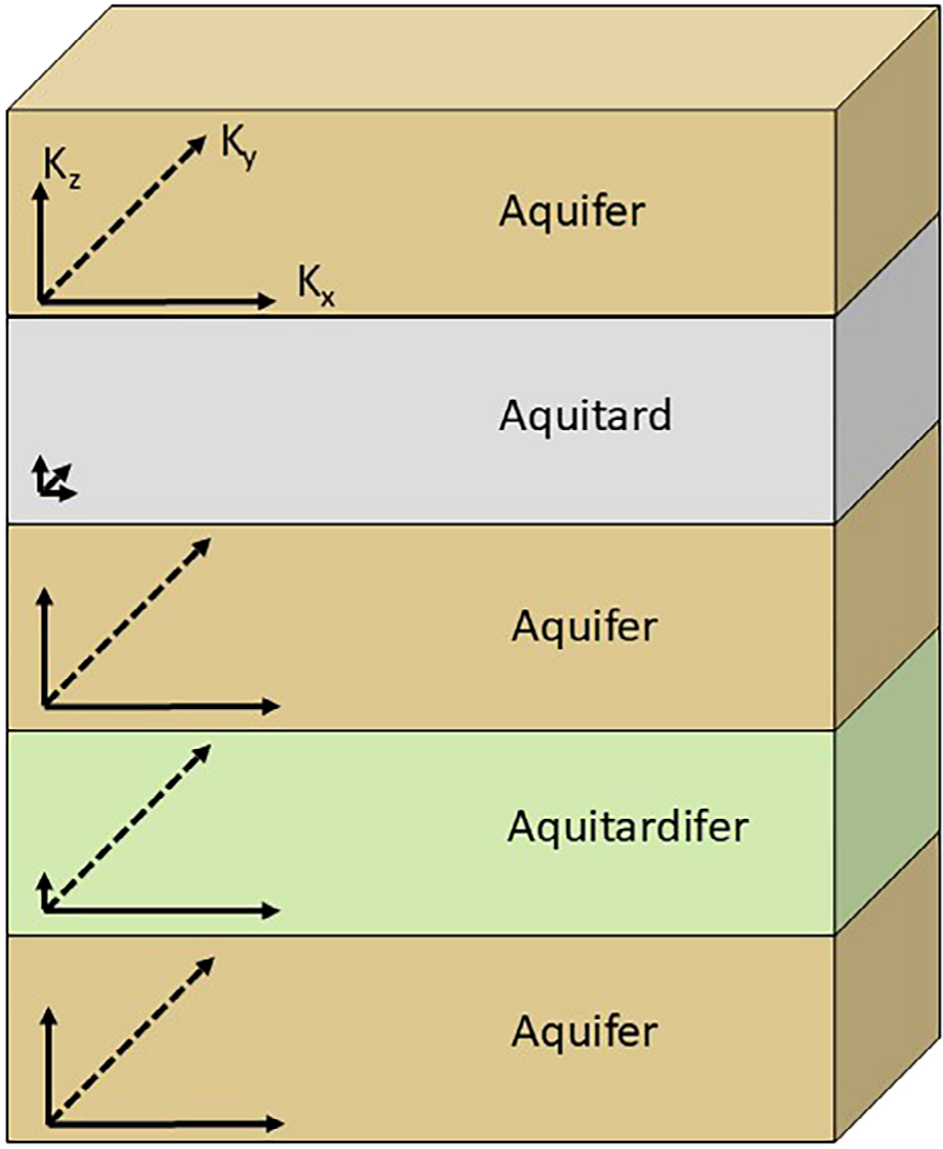
A multilayer hydrogeologic system with generalized two‐dimensional hydraulic conductivity vectors depicting the properties of an aquitardifer in comparison to typical aquifers and aquitards.

In this contribution, we elaborate on these and several other examples where the conventional terms aquifer and aquitard are inadequate to account for the properties of some geologic units. These examples suggest that such units should be expected to be common and justify the need for a new term, aquitardifer (Figure 1), to be added to the hydrogeologic nomenclature. Our definition of an aquitardifer is: *An anisotropic body of geologic material with permeability in at least one direction sufficiently lower than adjacent units such that it significantly impedes groundwater flow between units, but with permeability in other direction(s) sufficient to transmit water to wells and springs in quantities comparable to aquifers in the same system*. A geologic unit previously classified as an aquitardifer can be subsequently subdivided into aquifers, aquitards, and aquitardifers at a finer scale if sufficient information can eventually be acquired to do so at a practical scale.

## Key Examples

Here we summarize the results of three detailed studies of parts of the COAS of midcontinent North America. This aquifer system consists of largely undeformed, horizontal layers of sandstone, shale, and dolostone, traditionally divided into multiple hydrogeologic units classified as aquitards and aquifers. These three examples demonstrate, in different ways, the need for the term aquitardifer in hydrogeologic nomenclature.

### St Lawrence Aquitardifer

**Figure 2:**
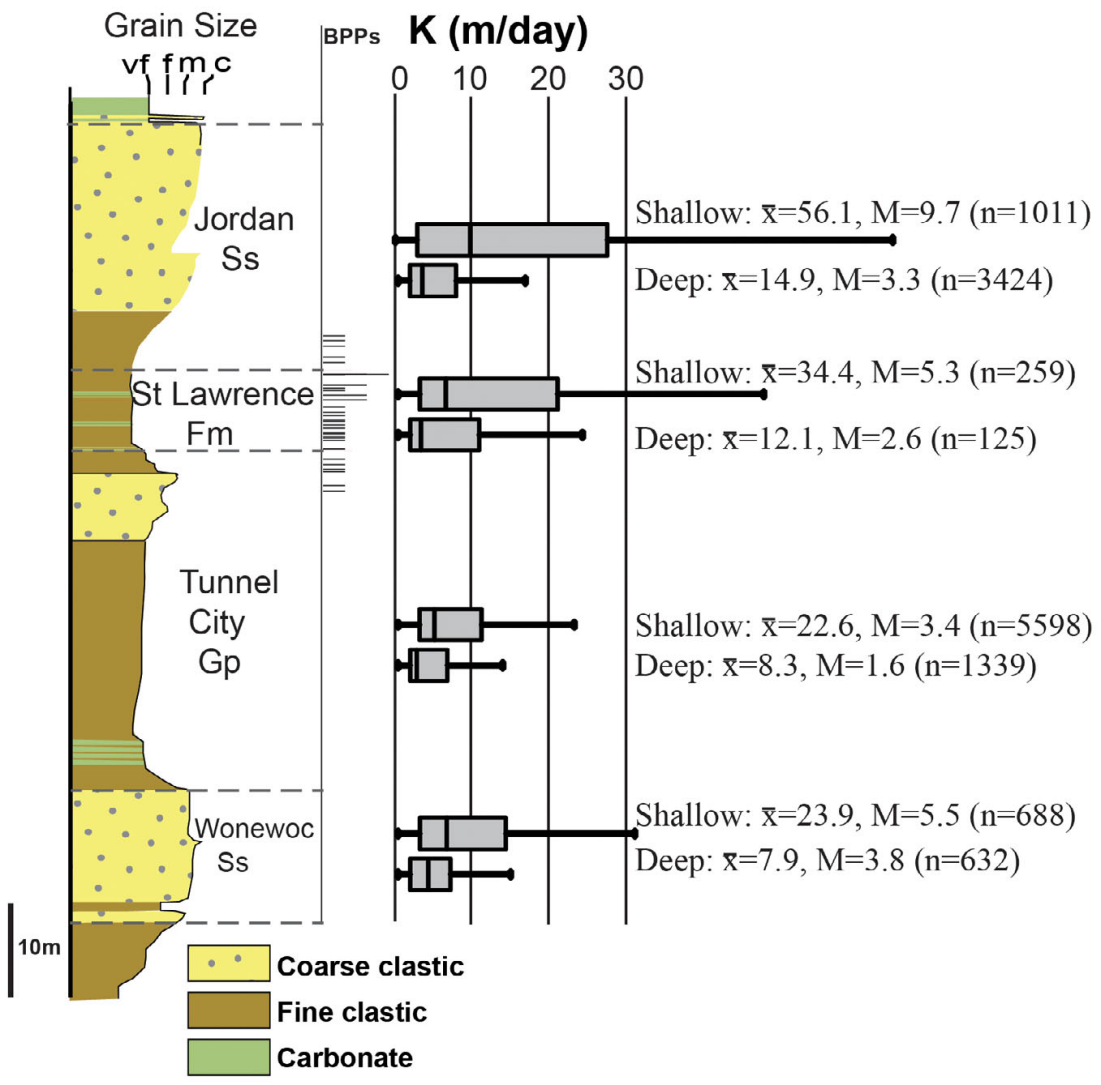
Boxplots of horizontal hydraulic conductivity (*K*) data for 13,076 wells open to Cambrian Wonewoc Sandstone, Tunnel City Group, St. Lawrence Formation, and Jordan Sandstone in southeastern Minnesota. The hydraulic conductivity values for each formation are subdivided into wells open to shallow bedrock conditions (open hole within 15 m of bedrock surface) and deep bedrock conditions (well cased below 15 m from bedrock surface). Note that the hydraulic conductivity of the St. Lawrence Formation, which has only low‐permeability matrix (fine clastic and carbonate), is generally similar to that of the other units, which contain intervals of coarse clastic sandstone with high matrix permeability. x, average; M, median; n, number of values. The stratigraphic positions of hydraulically active bed parallel partings (BPPs) are based on ambient flow noted in 13 boreholes with geophysical logs (i.e., flowmeter, video, and fluid conductivity and temperature) in southeastern Minnesota (Runkel et al. 2014). Vertical scale is approximate, as these formations vary in thickness. Modified from Runkel et al. (2018).

The Cambrian St. Lawrence Formation in southeastern Minnesota is a ~8 to 28 m thick, fractured, heterolithic, siliciclastic‐dominated bedrock layer with low matrix permeability (Kv median values 10^−4^ to 10^−7^ m/day) (Runkel et al. 2018). It is traditionally classified as an aquitard (or in prior usage “confining unit”), which is consistent with studies showing that across some areas young water impacted by human activity is separated from older, unimpacted water at depths that approximate the position of the St. Lawrence Formation (Berg 2003; Petersen 2005; Tipping 2012). However, the St. Lawrence aquitard has also been shown to have a Kh comparable to units traditionally classified as aquifers in the COAS (Figure 2). It is therefore widely used as a source of water across southeastern Minnesota, with over 900 known wells (County Well Index 2025), and there are over 350 mapped springs that emerge from the St. Lawrence, commonly with discharge of several liters or more per minute (Runkel et al. 2018).

**Figure 3:**
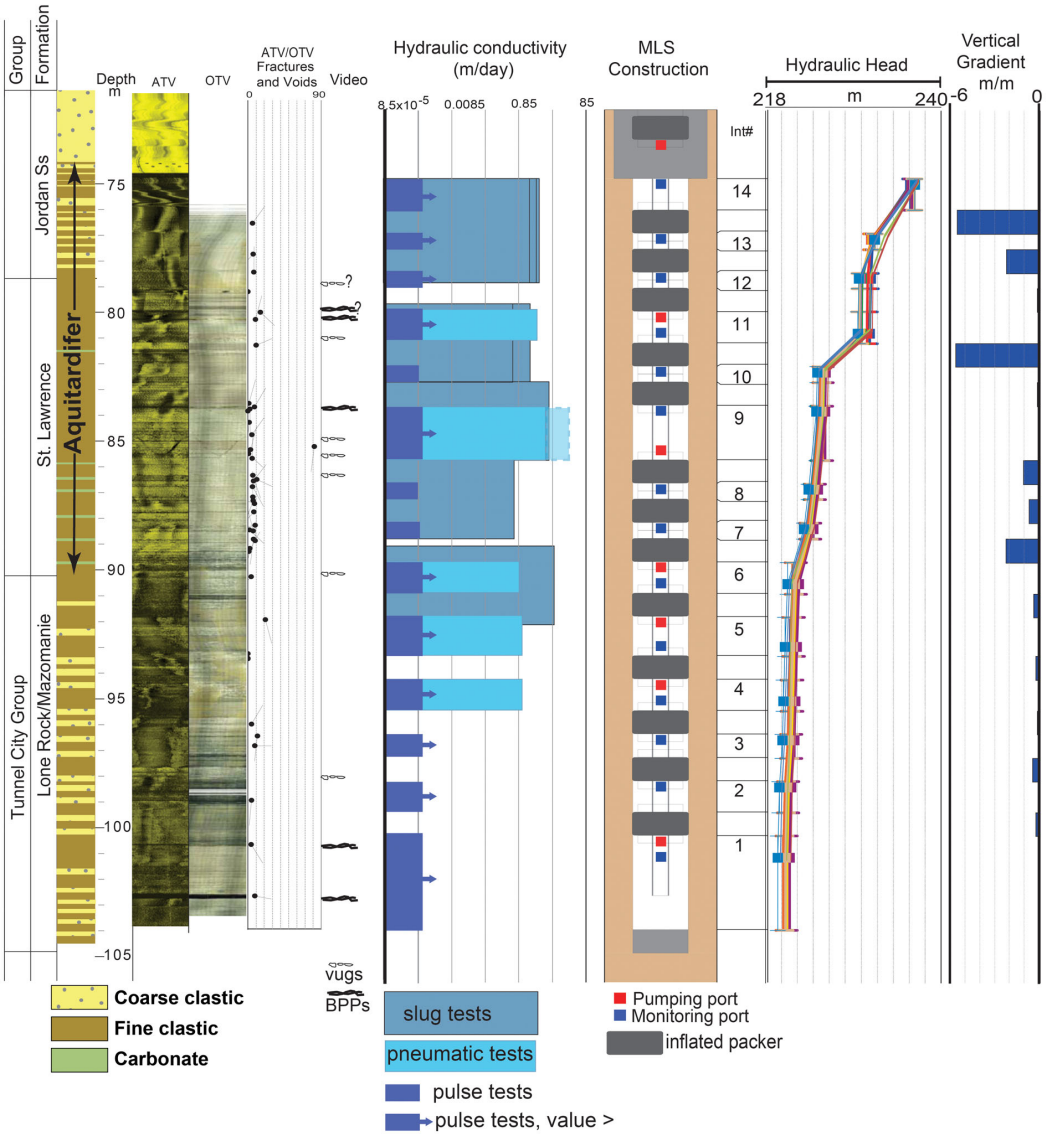
Lithologic, borehole geophysical, and hydraulic test data from a borehole ultimately instrumented with a multilevel monitoring system. Horizontal hydraulic conductivity was measured via slug tests in 10‐foot packed intervals, as well as slug tests across 14 0.6 m intervals using the pulse method, and across five intervals ranging from 0.6 to 3 m with a pneumatic method. The three columns on the far right show the position of packers and monitored zones with pumping and measurement ports, and hydraulic head data. The intervals with large vertical gradients are inferred to be of particularly low vertical hydraulic conductivity relative to the adjacent units (Meyer et al. 2014). Methods and additional results in Runkel et al. (2018). The results for monitoring intervals with K that was too high (>10^−3^ m/day) for the pulse test method are shown as minimum values, with an arrow. ATV, acoustic televiewer; OTV, optical televiewer. Modified from Runkel et al. (2018).

Runkel et al. (2014, 2018) used multidisciplinary techniques including borehole flowmeter logging, high‐resolution depth‐discrete multilevel well monitoring, packer testing, fracture stratigraphy, fluorescent dye tracing, and three‐dimensional (3D) distribution of anthropogenic tracers regionally across southeast Minnesota to better understand the seemingly paradoxical characteristics of the St Lawrence Formation and adjacent strata. They showed that across most of southeast Minnesota the St Lawrence Formation (along with the overlying lower part of the Jordan Sandstone) is indeed a high‐integrity aquitard, as conventionally defined, because it contains low vertical hydraulic conductivity (Kv) intervals where vertical fractures are poorly connected. In fact, low Kv intervals can be present at several positions within the lower Jordan‐St Lawrence, as shown by the vertical gradients (Figure 3), and based on the variable stratigraphic positions of ambient flow between BPPs in numerous boreholes, as well as those of preferential termination horizons (PTHs) of vertical fractures in outcrops (Runkel et al. 2014, 2018). Matrix Kv can be as low as 10^−9^ m/day, and bulk, field‐scale Kv for the St Lawrence was estimated at about 10^−3^ to 10^−4^ m/day (Runkel et al. 2018). Borehole logging and field examination of springs showed that the high bulk Kh of this same interval is largely due to ubiquitous bed parallel partings (BPPs), which also occur at variable stratigraphic positions (Figures 2 and 3). Because of these characteristics, it is impractical to divide the lower Jordan‐St Lawrence interval into discrete aquifers and aquitards. Instead, because the interval can be properly classified as both an aquitard and an aquifer, even in a single location, its properties are best accounted for by classification as an aquitardifer (Runkel et al. 2018).

### Tunnel City Aquitardifers

**Figure 4:**
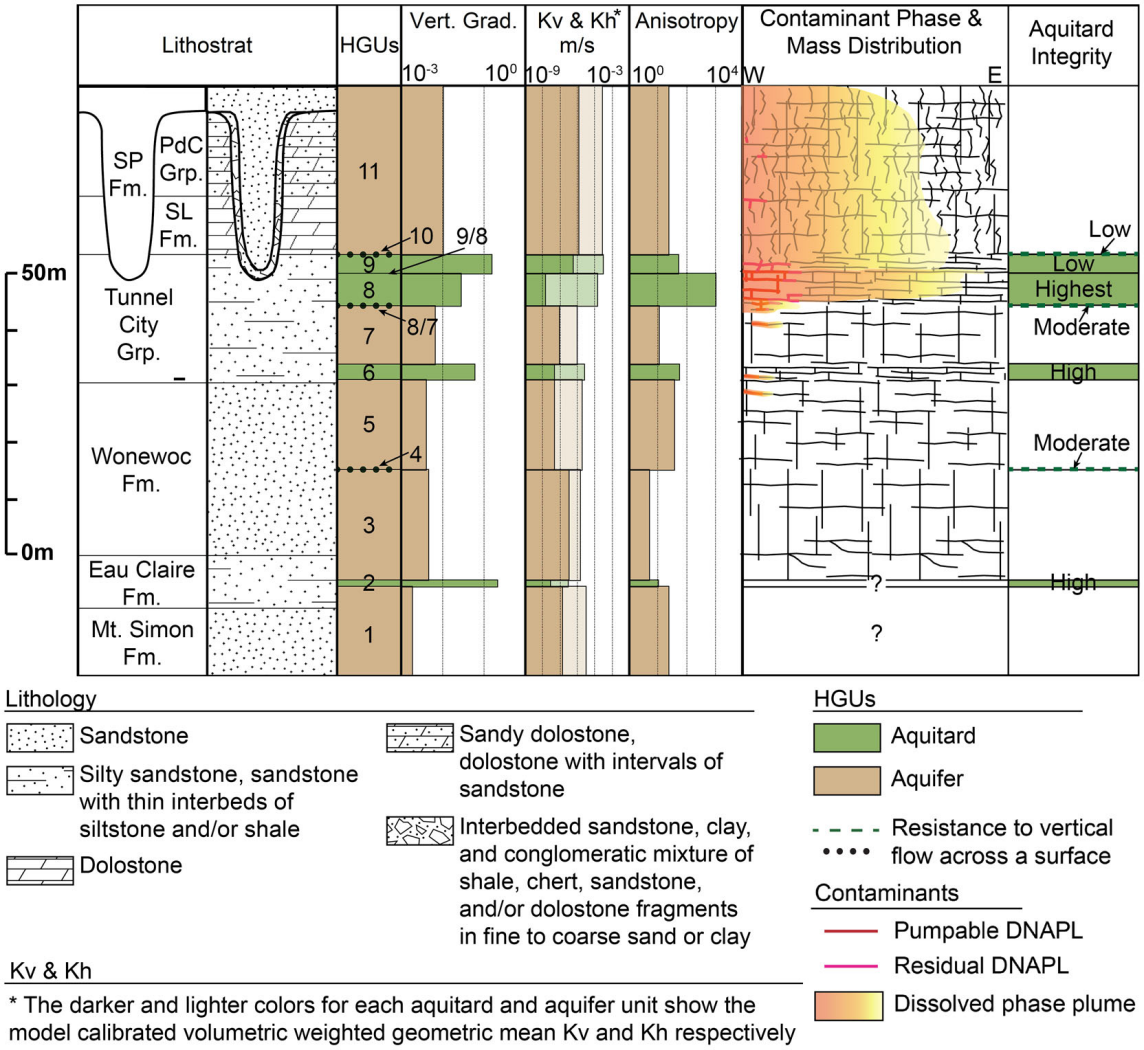
Conceptual model depicting the geologic and hydrogeologic characteristics of Cambrian bedrock aquitards identified at a DNAPL site in southeastern Wisconsin. The observed integrity of the shallow bedrock and surface 10, HGU9, and HGU8 aquitards to DNAPL migration and the inferred integrity of each of the deeper aquitard units are shown. Note that the Kh of even the high‐integrity aquitards is as great as or even greater than the aquifers. Modified from Meyer et al. (2023).

Extensive characterization of a DNAPL site in southeastern Wisconsin (Meyer et al. 2008, 2016, 2023) provided exceptional insights into the delineation of hydrogeologic units in fractured, siliciclastic dominated sedimentary bedrock of Cambrian and Ordovician age. Their results indicated that traditional aquitard and aquifer delineation was not sufficient to capture all of the hydraulic contrasts important to flow and contaminant transport. High‐resolution vertical hydraulic head profiles and sequence stratigraphy were used to delineate intervals and surfaces with lower Kv (Meyer et al. 2008, 2016), followed by a study that used pre‐existing DNAPL contamination as a tracer to independently identify aquitards within the upper part of the aquifer system and assess the integrity of those aquitards (Meyer et al. 2023). Those hydrogeologic units were used as the framework for a numerical model that was calibrated using the high‐resolution head profiles, providing well‐constrained estimates of Kh and Kv. They combined the insights from the development of the hydrogeologic framework, the contaminant distribution, the modeling results, and fracture network data to assess the aquitard characteristics (vertical gradients, Kv, anisotropy, vertical fracture connectivity) within the regionally important aquifer system (Figure 4).

Units considered to be aquitards at the site are relatively thin and highly anisotropic. Vertical hydraulic conductivity is 1 to 3 orders of magnitude less than Kh, with the latter commonly similar to or even greater than the Kh values for units designated as aquifers (Meyer et al. 2023). The anisotropy of the aquitards can be attributed to laterally extensive BPPs coupled with short, frequently terminating, and poorly connected vertical fractures. An 8 to 9 m thick aquitard that plays a key role in limiting downward transport of contaminants, HGU8, exemplifies the anisotropic character of the aquitards at the site, rendering classification using traditional terms of aquifer or aquitard challenging. It has an anisotropy factor of 1000, with a geometric mean Kh value higher than all but one of the aquifers. Yet HGU8 has the highest aquitard integrity. Although DNAPL penetrated into HGU8, the unit had a large capacity to accumulate/store DNAPL phase in the BPPs. The relatively low Kv and large anisotropy of the unit also minimized downward spread of the dissolved phase plume and promoted lateral transport (Meyer et al. 2023).

### Platteville Aquitardifer

Research on the Platteville Formation in the Twin Cities Metropolitan area (TCMA) (Anderson et al. 2011; Runkel et al. 2015, 2019) provided detailed insights into anisotropic intervals that cannot be satisfactorily subdivided into aquifers and aquitards even at the submeter scale. The Platteville is an approximately 9 m thick, Upper Ordovician carbonate (dolostone and limestone) to shaly carbonate. Matrix porosity and permeability are very low (<10^−7^ m/day; Runkel et al. 2015) and it has been conventionally classified as an aquitard. It has been investigated for decades as part of remediation efforts at a large number of contaminated sites in the TCMA, which have locally provided support for aquitard delineation by revealing measurable and mappable vertical head gradients within the Platteville (Barr Engineering 1983). However, subvertical fractures and especially BPPs accommodate yields sufficient for the Platteville to be used as a source of water, with 732 known domestic wells (County Well Index 2025), and packer tests have documented Kh values akin to those of aquifers in the region (Runkel et al. 2015). The Platteville is also the source of dozens of springs along the Mississippi River and its tributaries (Brick 1997; Anderson et al. 2011; DNR 2025). The apparent discrepancy whereby the Platteville has properties consistent with classification as an aquitard yet yields significant amounts of water led to our earliest suggestion that some geologic materials have properties that are a “hybrid” of aquitards and aquifers (Runkel 2010; Anderson et al. 2011).

**Figure 5:**
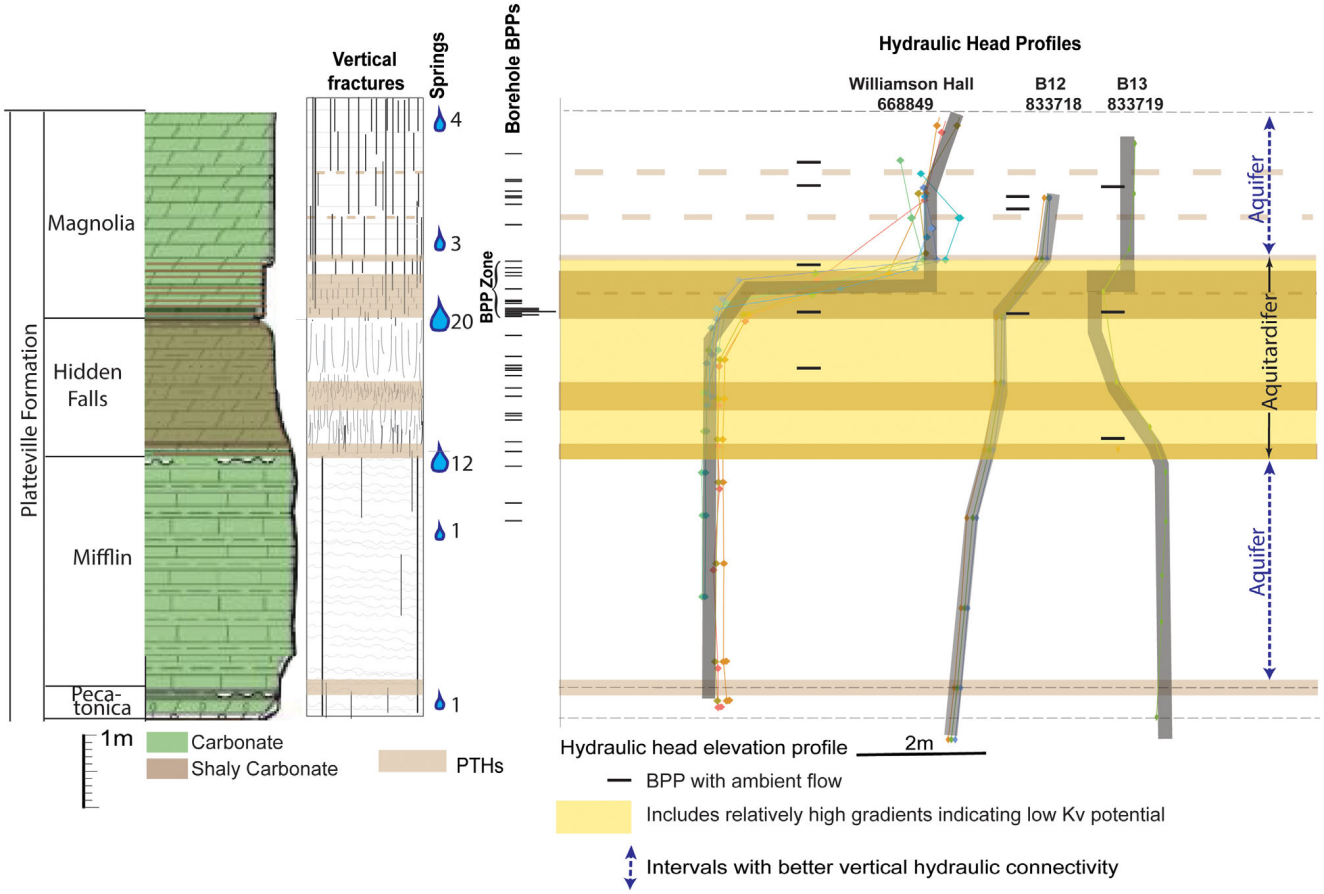
Generalized stratigraphic column showing the lithology, thickness, and nomenclature for the Platteville Formation in the TCMA, along with key hydrogeologic properties. Schematic depiction of vertical fractures and their preferential termination horizons (PTHs) is based on outcrops. The spring count lists the number of springs emanating from a specific stratigraphic interval. The borehole BPPs shown are ones through which ambient or induced flow occurred based on borehole geophysical logs from a total of 18 monitor wells. The vertical hydraulic head profiles, also showing hydraulically active BPPs, are from three wells at two sites about 7.1 km from one another. Modified from illustrations in Runkel et al. (2019). Vertical scale is approximate because unit thicknesses vary slightly across the TCMA.

In an effort to provide a more robust hydrogeologic conceptual model for the Platteville Formation in the TCMA, Runkel et al. (2019) synthesized information from outcrop fracture mapping, high resolution vertical hydraulic head profiles, packer tests, flowmeter logs, and stratigraphic positions of springs. A summary of key results in Figure 5 shows that at the scale of its entire 9 m thickness, the formation can be divided into two aquifers separated by an ~2.7 m interval most appropriately classified as an aquitardifer. The aquitardifer corresponds to shale‐rich carbonate beds of the Hidden Falls Member and lowermost Magnolia and uppermost Mifflin members strata immediately above and below it. It contains at least three potential low Kv intervals based on fracture terminations and vertical hydraulic head inflections and also has hydraulically active BPPs at multiple stratigraphic positions, including within the low Kv intervals. Seventy‐five percent of mapped Platteville springs emanate from this 2.7 m interval, and bulk Kh based on packer tests is commonly a few to tens of m/d (Runkel et al. 2015).

**Figure 6:**
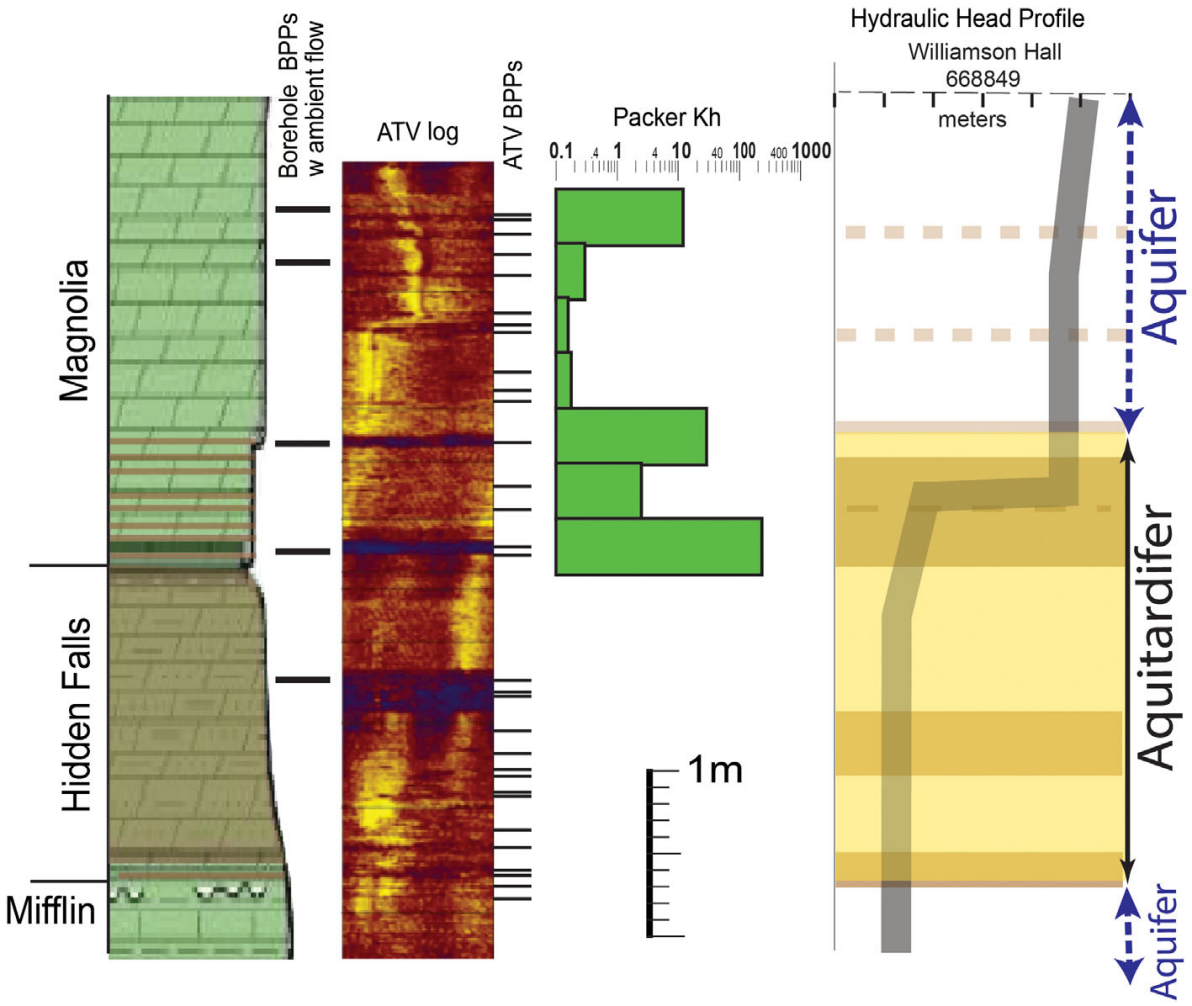
Acoustic televiewer (ATV) image, discrete interval horizontal hydraulic conductivity, and vertical hydraulic head profile for a monitor well on the University of Minnesota campus. ATV BPPs include aligned voids. Horizontal hydraulic conductivity is based on 1‐foot interval packer tests.

The highest resolution of hydraulic properties acquired by Runkel et al. (2019) is across ~70 to 80 cm of the lowermost Magnolia and uppermost Hidden Falls members, which is best classified as an aquitardifer *even at this scale*. This interval corresponds to the largest hydraulic head inflection in each of the instrumented boreholes (Figure 5), indicating lower Kv than adjacent units. It also corresponds to the highest density of hydraulic BPPs, which we refer to as a “BPP zone” Nearly 50 percent of Platteville springs emerge from this thin interval, with flow rates as high as 473 L/min (Anderson et al. 2011). Ambient flow through individual BPPs in boreholes is commonly measured in several liters per minute, and injection flowmeter logging shows that Kh in discrete cm‐scale intervals with one or more BPPs is commonly 10's to 1000's m/day (Runkel et al. 2015, 2019). Thirty‐centimeter interval packer tests across this interval, where it includes a strong vertical head gradient of 3 m/m, documented Kh that ranges from 0.7 m/day to 8.6 m/day (Figure 6). Even the minimum value is higher than the Kh across much of the overlying Magnolia that is considered to be an aquifer based on the low vertical hydraulic head gradient. Borehole flowmeter and acoustic televiewer logs show that the relatively high Kh is via the presence of BPPs, perhaps enhanced to some extent by the dissolution of carbonate.

The most practical subclassification of the Platteville would include applying the term aquitardifer to the 2.7‐m interval that contains all known potential low Kv intervals and also has a high density of BPPs. Further subdivision of this aquitardifer into aquifers and aquitards is not possible for a number of reasons. The submeter low Kv intervals have high hydraulic conductivity BPPs within and along their boundaries (Figures 5 and 6). Although our ongoing work indicates it may be feasible to acquire cm scale, depth discrete hydraulic head measurements that could discriminate low Kv interval(s) that lie between BPPs in an individual borehole, the applicability to other areas at this detailed resolution would be questionable because the stratigraphic position of BPPs is variable from place to place within this thin interval (Figure 5), and there are subtle changes in bedding that may cause variability in the positions of PTHs of vertical fractures that correspond to low Kv horizons. Furthermore, not all vertical hydraulic head inflections indicative of low Kv are consistently present at precisely the same stratigraphic positions, nor do all PTHs have head inflections in all three of the instrumented wells. This allows for the possibility that some of the PTHs are not laterally continuous. Conversely, there may be more low Kv intervals within the Platteville Formation than recognized, because the absence of hydraulic head inflections does not necessarily indicate the absence of a low Kv interval (e.g., Meyer et al. 2014, 2016). An additional complexity is that the low Kv intervals in the Platteville, as well as other units classified as aquitards, commonly do not extend to eroded edges of the aquitards where vertical fractures are more fully connected (Runkel et al. 2018, 2019).

## Other Examples

Here we provide additional examples of geologic materials having properties such that classification as only an aquitard or aquifer appears to be unsatisfactory. Included are additional examples of siliciclastic and carbonate sedimentary bedrock with low matrix hydraulic conductivity and BPPs, as well as unlithified to poorly lithified heterogeneous sediment.

### Siliciclastic Bedrock

Peffer (1991), provides an excellent early example demonstrating the terms aquifer and aquitard are insufficient to classify some hydrogeologic systems. Peffer (1991) used hydrographs, vertical gradients, ground‐water chemistry, borehole geophysical logs, and pumping tests to document high vertical to horizontal anisotropy in the Pennsylvanian Clarion Shale at a site in the Appalachian Plateau in southwest Pennsylvania, USA. Over 15 m of head loss and substantial groundwater quality differences across the ~21 m thick shale demonstrated at least parts of it are of low Kv. Yet the Clarion Shale yields in wells were as high as 227 L/min, produced via thin brittle interbeds and bedding partings that imparted a high bulk Kh. Given these properties, Peffer (1991) suggested that the Clarion Shale “…performed in some ways as an aquitard; yet it was capable of high well yields which suggested it was an aquifer.” He noted that “The simple concept of aquifers as units that support well yields, and aquitards as units that do not, is not easily applied in the Appalachian Plateau,” further suggesting that some shaly bedrock layers elsewhere in the plateau may be complex in that they can act as regionally extensive aquitards yet yield enough water to be considered an aquifer, at least locally.

Cherry et al. (2006) cites Peffer's (1991) example of sedimentary bedrock aquitards that can be highly anisotropic due to significant horizontal hydraulic conductivity and also highlight somewhat similar examples for the Triassic Passaic Formation in the Newark Basin (Michalski and Britton 1997), and the Ordovician Maquoketa Formation and Cambrian Tunnel City Group in Wisconsin. Cherry et al. (2006) suggested “The common assumption of primarily vertical flow in aquitards may not be appropriate for some lithified aquitards where complex flow paths influenced by bedding planes linking vertical fractures may be dominant (Eaton and Bradbury 2003).” And furthermore that “Because of such highly anisotropic fracture networks, silt‐ and sandstone formations considered aquitards in the context of vertical flow between major aquifers can be sources for springflow due to horizontal flow allowed by the much larger horizontal component of hydraulic conductivity (Swanson 2006).” Cherry et al. (2006) used these examples to emphasize the importance of distinguishing Kh from Kv as well as considering the scale and context at which a hydrogeologic unit is studied.

The investigation of the Maquoketa aquitard in Wisconsin by Eaton and Bradbury (2003) and Eaton et al. (2007) was particularly influential on our subsequent studies of aquitard properties. They used discrete interval head measurements, downhole geophysical logs, and estimates of hydraulic conductivity to show that the Maquoketa aquitard, while restricting downward ground water flow, has a complex internal flow system due to bedding‐plane fractures. They documented a very low Kv but also high Kh within the aquitard due to these fractures, which can allow significant lateral groundwater flow. They suggested that, in contrast to conventional hydrogeological conceptual models, flow in aquitards cannot always be inferred to be primarily one‐dimensional and vertical.

The Passaic Formation, where studied in the Newark Basin by Michalski (1990), and Michalski and Britton (1997), is largely mudstone and shale with low matrix permeability. They referred to it as an “aquifer system” characterized by bedding‐plane partings that are the principal groundwater flow pathways. Vertical hydraulic head data from conventional monitoring wells along with water chemistry suggest a “multi‐unit structure” whereby at least some intervals of bedding‐plane partings are separated by low Kv aquitards. They recommended that investigations going forward should target higher resolution identification of discrete water‐producing bedding‐plane partings and the low Kv aquitards that provide some degree of hydraulic separation between them (Michalski and Britton 1997). More recent remedial activities in the Newark Basin using borehole geophysics, discrete interval hydraulic head information, water chemistry, and outcrop observations of bed‐perpendicular fracture terminations have revealed a very close spatial relationship whereby the highest Kh flow zones are commonly within shaley beds that also have a low Kv. These circumstances of high Kh within discrete intervals of low Kv appear to be similar to the properties of aquitards in the COAS documented by Meyer et al. (2016, 2023) and Runkel et al. (2018, 2019) described in this report, and have led the project investigators to suggest that the term aquitardifer best characterizes these intervals at their site in the Newark Basin (Mastera 2023, 2025). These conditions may also apply to other parts of the stratigraphic succession in the Newark Basin, such as the Lockatong and Stockton Formations, described by Lacombe and Burton (2010) as having stratabound vertical joints and discrete, thin intervals containing abundant BPPs.

### Carbonate Bedrock

Skinner (2019), Johnson (2020) and Medici et al. (2024) used methods similar to those of Meyer et al. (2016, 2023) by combining fracture network characteristics and associated vertical hydraulic head profiles from 24 cored holes to differentiate hydrogeologic units in a ~100‐m‐thick heterogeneous Silurian dolostone aquifer system (Lockport Group) that supplies water to the city of Guelph, Ontario, Canada. They recognized four aquitards that are laterally discontinuous (“patchy”), at variable depths and stratigraphic positions, and controlled largely by the frequency, height, and termination characteristics of high‐angle fractures. Packer tests showed that one of the aquitards (HGU9), a 2.5 to 9 m interval within the marl‐rich Vinemount Member, has higher Kh than all but one of the designated aquifers (Skinner 2019; Johnson 2020) due to interconnected secondary pores. Thus, HGU9 has adequate Kh to be classified as an aquifer, even though the Kv is low enough to be classified as an aquitard.

### Unlithified to Poorly Lithified Sediment

Although the best documented and most numerous examples of geologic units with both aquifer and aquitard properties appear to be for sedimentary bedrock, common facies of unlithified sediment can be similarly anisotropic and challenging to classify using conventional hydrogeologic terms. In contrast to our examples of sedimentary bedrock, which rely on the presence of bed parallel partings, thin interbeds of low and high permeability unlithified sediments at a scale whereby individual aquifers and aquitards cannot be practically differentiated suggest the need for a term such as aquitardifer. Beyond the examples provided below, Shepley (2024) summarizes a large number of additional examples of sediment with highly anisotropic properties that could be considered consistent with classification as an aquitardifer.

The term aquitardifer could be applied to thinly interbedded sand, silt, and clay, a common facies in a number of depositional environments such as alluvial overbank, tidal, deltaic, and lacustrine systems (e.g., Bhattacharya 2010; Dalrymple 2010; Miall 2010; Renaut and Gierlowski‐Kordesch 2010). An example are the unconsolidated littoral spit deposits that are part of a “surficial aquifer” in Sainy‐Lambert‐de‐Lauzon, Quebec, Canada, studied by Paradis and Lefebvre (2013). Grain sizes range from fine sand to clayey silt, with rapid transitions in grain size over less than a decimeter. Vertical profiles of hydraulic conductivity and hydraulic heads indicate “semi‐confined” conditions, which the authors interpreted to be the result of the alternation of sand and silt layers. Vertical interference slug tests (61 cm intervals) along with field and laboratory measurements of Kh and Kv, revealed anisotropy as high as 3 orders of magnitude. Kh was commonly 10^−5^ m/s, and Kv 10^−7^ to 10^−8^ m/s, values within the range of aquifers and aquitards, respectively, as traditionally defined.

Sedimentary interbeds that are part of the Miocene Columbia Plateau Regional Aquifer System (Washington, Oregon, Idaho) may be best classified as aquitardifers. These sedimentary deposits as well as dense interiors of individual basalt flows act as aquitards (Reidel et al. 2002) imparting a bulk horizontal to vertical anisotropy in the aquifer system as high as 1000:1 based on model calibrations (Whiteman et al. 1994; Kahle et al. 2011). Individual interflow sedimentary units believed to be acting as aquitards could be considered aquitardifers because they are commonly transmissive enough in a horizontal direction to “function as aquifers” (Whiteman et al. 1994) due to the presence of permeable sand and sandstone interbeds. Even two of the most well‐documented and laterally extensive “confining units” in the Regional Aquifer System, the Saddle Mountains‐Wanapum and Wanapum‐Grande Ronde interflow units, have properties consistent with an aquitardifer classification. Whiteman et al. (1994) note they are not formally named as confining units in part because at some locations they consist of materials that are at least as permeable as the basalts. Basalt aquifer packages separated by these two “confining units” also contain sedimentary interbeds believed to serve as aquitards of more limited lateral extent, and they too can transmit enough water to serve as aquifers (Whiteman et al. 1994).

## Aquitardifer Justification, Definition, and Applicability

Hydrogeologic nomenclature is important, as it often determines how a geologic unit is treated in regulatory codes such as those governing water well construction, as well as how the unit is characterized in site conceptual models that guide numerical modeling and other methods used to predict contaminant transport and water budgets. As noted by Van der Gun (2022), hydrogeologic literature includes many different interpretations of the aquifer concept, with purposefully vague definitions. Among the many definitions compiled by Van der Gun (2022) there is however a consistent theme whereby an aquifer is a body of geologic material of sufficient permeability to yield water (to wells or springs) in significant (aka “exploitable,” “usable,” “economic”) amounts. The definitions of aquitard also vary but typically characterize them as low permeability units that, in contrast to aquifers, do not yield significant amounts of water. For example, Woessner and Poeter (2020) describe aquitards as **“**units that store water and are less permeable than aquifers, so they slow the transmission of water. They may transmit water in regional layered flow systems where leakage passes through them from one aquifer to an overlying or underlying aquifer, yet their hydraulic conductivity is low enough that they cannot produce useful quantities of water”. More recently, Sharp Jr. (2023) defines an aquitard as “a geologic material, stratum, or formation of low permeability (a confining unit) that transmits significant amounts of water only at a regional scale or over geologic time”. Rarely, however, has it been acknowledged that some aquitards can transmit significant quantities of water. In making a distinction between aquifers and aquitards, Cherry et al. (2006) qualifies the water yielding properties of aquitards by suggesting “Aquitards, unlike aquifers, do not *generally* [emphasis added] supply economic quantities of water to wells”. Similarly, Anderson et al. (2015), note that while aquitards typically have low permeability, it is not uncommon for them to have fractures that impart significant permeability, citing one example of our informal use of the term aquitardifer (Green et al. 2012) to account for such units.

Quantification of the hydraulic properties of traditionally defined aquifers and aquitards does not clarify the distinction between the two because their ranges of hydraulic conductivity overlap (e.g., Woessner and Poeter 2020; figure 32). Cherry et al. (2006; tables 3.1 and 3.2) summarize a large number of hydraulic conductivity values for aquitards, and even Kv values for aquitards overlap with the Kh values for aquifers. These overlapping ranges are expected given the vague definitions of aquifer and aquitard and because the “significance” of yield to wells and springs depends on water availability in any given area. The overlap can also reflect the fact that contrast in K between an aquifer and aquitard, rather than the actual values of K, is critical in dictating flow (Freeze and Witherspoon 1967).

The hydrogeologic characteristics of geologic materials such as those described in this paper cannot be satisfactorily classified using currently available terminology. We suggest it is common that units conventionally classified as aquitards can accommodate significant flow, including yields to wells and springs in amounts analogous to units conventionally defined as aquifers. Conversely, it is common for units classified as aquifers to contain low Kv intervals that can act as aquitards in the conventional sense. Most stratified deposits (e.g., sedimentary and layered extrusive igneous rocks) are anisotropic, commonly with orders of magnitude greater hydraulic conductivity in a direction parallel to bedding than perpendicular to bedding (e.g., see Shepley (2024) for many examples). The examples of unlithified anisotropic sediment we provide herein could also readily apply to lithified sedimentary bedrock containing similar facies of thinly interbedded coarse‐ and fine‐grained material, even without BPPs, in instances where the coarse‐grained interbeds have retained much of their primary permeability. Furthermore, the presence and importance of hydraulically significant BPPs in sedimentary bedrock with low matrix permeability, long recognized in carbonate bedrock, have been increasingly documented in siliciclastic bedrock. For example, research on unconventional oil and gas reservoirs has commonly revealed BPPs in otherwise tight sandstone (e.g., Zeng et al. 2023) and shale (e.g., Gale et al. 2014), even at depths greatly exceeding those of most utilized aquifers. This emphasizes the need for wider deployment of multidisciplinary, high‐resolution techniques such as those summarized in this report, which is likely to lead to more widespread identification of aquitardifers. If information is insufficient, or it is impractical to differentiate individual aquitards and aquifers within highly anisotropic geologic materials, classification as only one of these two conventional options can obscure the presence of the other, which could be counterproductive to groundwater management and research. Classification as an aquifer because it yields significant quantities of water in a horizontal direction obscures the presence of low Kv intervals that can control flow and limit vertical migration of contaminants, with transport predictions compromised as a result. It can also lead to construction of wells with screens (and open holes) penetrating low Kv interval(s) that otherwise protect adjacent aquifers, cross‐connecting contaminated and uncontaminated groundwater. Conversely, classification as an aquitard on the basis of low matrix permeability, large vertical head gradients, or stratified water chemistry obscures the presence of high hydraulic conductivity in another direction that could be sufficient to provide economic quantities of water to wells and springs, and allow for significant contaminant transport. Classification as an aquitardifer instead of aquifer or aquitard will emphasize that highly anisotropic units can be key intervals where downward transport of contaminants is hindered, but lateral transport is promoted (Meyer et al. 2023).

Given the applied value in recognizing that many types of geologic materials may have properties of both aquitards and aquifers as traditionally defined, we propose that the term aquitardifer be added to the hydrogeologic nomenclature. Aquitardifer: *An anisotropic body of geologic material with permeability in at least one direction sufficiently lower than adjacent units such that it significantly impedes groundwater flow between units, but with permeability in other direction(s) sufficient to transmit water to wells and springs in quantities comparable to aquifers in the same system*. A geologic unit previously classified as an aquitardifer can be subsequently subdivided into aquifers, aquitards, and aquitardifers at a finer scale, if sufficient information can eventually be acquired to do so at a practical scale.

Although the examples of aquitardifers described herein refer to anisotropy whereby Kh is orders of magnitude greater than Kv, our definition intentionally excludes reference to such coordinates. In more structurally complex settings where strata are tilted, the strongest anisotropy will not necessarily be circumstances where Kh>Kv. For example, subvertical strata as well as faults (Caine et al. 1996; Bense and Person 2006) may have properties similar to the aquitardifers described herein, but with anisotropy whereby Kv greatly exceeds Kh. However, aquitards are also typically conceptualized as impeding vertical flow between aquifers and as protecting, to varying degrees, underlying aquifers from surficial contamination. And, although these units would share a similar style of anisotropy and may inhibit lateral flow (Bense and Person 2006), they would not provide underlying aquifers with protection from surficial contaminants, creating some ambiguity in the application of the term aquitardifer to represent these units.

## Data Availability

The data that support the findings are in a large number of already published reports and journal articles.

## References

[gwat70022-bib-0001] Anderson, M.P. , W.W. Woessner , and R.J. Hunt . 2015. Applied Groundwater Modeling: Simulation of Flow and Advective Transport, 564. San Diego, California: Academic Press.

[gwat70022-bib-0002] Anderson, J.R. , A.C. Runkel , R.G. Tipping , K. Barr , and E.C. Alexander Jr. 2011. Hydrostratigraphy of a fractured urban aquitard. In Geological Society of America Field Guides 24, 457–475. Boulder, Colorado: Geological Society of America. 10.1130/2011.0024(22)

[gwat70022-bib-0003] Barr Engineering . 1983. Site characterization study and remedial action plan, General Mills solvent disposal site, June, 1983. On file at the Minnesota Pollution Control Agency.

[gwat70022-bib-0004] Bense, V.F. , and M.A. Person . 2006. Faults as conduit‐barrier systems to fluid flow in siliciclastic sedimentary aquifers. Water Resources Research 42, no. 5: 1–18. 10.1029/2005WR004480

[gwat70022-bib-0005] Berg, J.A. 2003. Hydrogeologic Cross Sections, Plate 8, 1:100,000. Falteisek, J (project manager) Geologic Atlas of Goodhue County, Minnesota. Minnesota Department of Natural Resources, Division of Waters County Atlas C‐12, Part B.

[gwat70022-bib-0006] Bhattacharya, J.P. 2010. Deltas. In Facies models 4, ed. N.P. James , and R.W. Dalrymple , 233–264. St. John's: Geological Association of Canada.

[gwat70022-bib-0007] Brick, G. 1997. Along the great wall: Mapping the springs of the twin cities. Minnesota Groundwater Association Newsletter 16, no. 1: 1–7. https://www.mgwa.org/newsletter/mgwa1997‐1.pdf (accessed March, 2025).

[gwat70022-bib-0008] Caine, J.S. , J.P. Evans , and C.B. Forster . 1996. Fault zone architecture and permeability structure. Geology 24, no. 11: 1025–1028.

[gwat70022-bib-0009] Cherry, J.A. , B.L. Parker , K.R. Bradbury , T.T. Eaton , M.B. Gotkowitz , D.J. Hart , and M.A. Borchardt . 2006. Contaminant Transport through Aquitards: A State‐of‐the‐Science Review, 126. Denver, Colorado: AWWA Research Foundation.

[gwat70022-bib-0010] County Well Index . 2025. Database created and maintained by the Minnesota Geological Survey, University of Minnesota, with the assistance of the Minnesota Department of Health. http://www.health.state.mn.us/divs/eh/cwi/ (accessed 2025).

[gwat70022-bib-0011] Dalrymple, R.W. 2010. Tidal depositional systems. In Facies Models 4, ed. N.P. James , and R.W. Dalrymple , 201–231. St. John's: Geological Association of Canada.

[gwat70022-bib-0012] DNR . 2025. Minnesota Spring Inventory: Minnesota Department of Natural Resources, Groundwater Atlas Program, statewide dataset of springs. https://www.dnr.state.mn.us/waters/groundwater_section/mapping/springs‐msi.html (accessed April 2025).

[gwat70022-bib-0013] Eaton, T.T. , M.P. Anderson , and K.R. Bradbury . 2007. Fracture control of ground water flow and water chemistry in a rock aquitard. Groundwater 45, no. 5: 601–615. 10.1111/j.1745-6584.2007.00335.x 17760586

[gwat70022-bib-0014] Eaton, T.T. , and K.R. Bradbury . 2003. Hydraulic transience and the role of bedding fractures in a bedrock aquitard, southeastern Wisconsin, USA. Geophysical Research Letters 30, no. 18: 1961. 10.1029/2003GL017913

[gwat70022-bib-0015] Freeze, R.A. , and P.A. Witherspoon . 1967. Theoretical analysis of regional groundwater flow; [part] 2, effect of water‐table configuration and subsurface permeability variation. Water Resources Research 3: 623–634. 10.1029/WR003i002p00623

[gwat70022-bib-0016] Gale, J.F.W. , S.E. Laubach , J.E. Olson , P. Eichhubl , and A. Fall . 2014. Natural fractures in shale: A review and new observations. AAPG Bulletin 98, no. 11: 2165–2216. 10.1306/08121413151

[gwat70022-bib-0017] Green, J.A. , A.C. Runkel , and E.C. Alexander Jr . 2012. Conduit flow characteristics of the St. Lawrence aquitardifer. In: Minnesota Ground Water Association Spring Conference Conduits, Karst, and Contamination April 19, 2012. http://www.mgwa.org/meetings/2012_spring/2012_spring_abstracts.pdf (accessed March, 2025).

[gwat70022-bib-0018] Johnson, K. 2020. High resolution dynamic pore pressure monitoring to determine hydraulic parameters in a multi‐layered bedrock system for improved aquifer vulnerability assessment and monitoring. MSc thesis, University of Guelph, Guelph, ON, Canada. https://atrium.lib.uoguelph.ca/server/api/core/bitstreams/c9b6b4f1‐5671‐4700‐aa22‐87c873d17176/content (accessed April, 2025).

[gwat70022-bib-0019] Kahle, S.C. , D.S. Morgan , W.B. Welch , D.M. Ely , S.R. Hinkle , J.J. Vaccaro , and L.L. Orzol . 2011. Hydrogeologic Framework and Hydrologic Budget Components of the Columbia Plateau Regional Aquifer System, Washington, Oregon, and Idaho. U.S. Geological Survey Scientific Investigations Report 2011‐5124: 66 p. 10.3133/sir20115124

[gwat70022-bib-0020] Lacombe, P.J. , and W.C. Burton . 2010. Hydrogeologic framework of fractured sedimentary rock, Newark Basin, New Jersey. Groundwater Monitoring & Remediation 30, no. 2: 35–45. 10.1111/j.1745-6592.2010.01275.x

[gwat70022-bib-0021] Mastera, L. 2025. Lawrence Mastera, Technical Consulting Director, Hydrogeology, ERM Sustainability Institute, copy of 2024 presentation sent to Anthony Runkel, 11, February.

[gwat70022-bib-0022] Mastera, L. 2023. Lawrence Mastera, Technical Consulting Director, Hydrogeology, ERM Sustainability Institute, email communication with Anthony Runkel, 6, October.

[gwat70022-bib-0023] Medici, G. , J.D. Munn , and B.L. Parker . 2024. Delineating aquitard characteristics within a Silurian dolostone aquifer using high‐density hydraulic head and fracture datasets. Hydrogeology Journal 32: 1663–1691. 10.1007/s10040-024-02824-9

[gwat70022-bib-0024] Meyer, J.R. , B.L. Parker , D.G. Abbey , S.G. Shikaze , L. Weaver , G. Merritt , L.A. Ribeiro , C.A. Morgan , and A.C. Runkel . 2023. Rock core VOC profiles diagnostic of aquitard occurrence and integrity in a multi‐layered sedimentary rock aquifer flow system. Journal of Hydrology 626: 130347. 10.1016/j.jhydrol.2023.130347

[gwat70022-bib-0025] Meyer, J.R. , B.L. Parker , E. Arnaud , and A.C. Runkel . 2016. Combining high resolution vertical gradients and sequence stratigraphy to delineate hydrogeologic units for a contaminated sedimentary rock aquifer system. Journal of Hydrology 534: 505–523. 10.1016/j.jhydrol.2016.01.015

[gwat70022-bib-0026] Meyer, J.R. , B.L. Parker , and J.A. Cherry . 2014. Characteristics of high resolution hydraulic head profiles and vertical gradients in fractured sedimentary rocks. Journal of Hydrology 517: 493–507.

[gwat70022-bib-0027] Meyer, J.R. , B.L. Parker , and J.A. Cherry . 2008. Detailed hydraulic head profiles as essential data for defining hydrogeologic units in layered fractured sedimentary rock. Environmental Geology 56: 27–44. 10.1007/s00254-007-1137-4

[gwat70022-bib-0028] Miall, A. 2010. Alluvial deposits. In Facies models 4, ed. N.P. James , and R.W. Dalrymple , 105–137. St. John's: Geological Association of Canada.

[gwat70022-bib-0029] Michalski, A. , and R. Britton . 1997. The role of bedding fractures in the hydrogeology of sedimentary bedrock—Evidence from the Newark Basin, New Jersey. Ground Water 35, no. 2: 318–327. 10.1111/j.1745-6584.1997.tb00089.x

[gwat70022-bib-0030] Michalski, A. 1990. Hydrogeology of the Brunswick (Passaic) formation and implications for ground water monitoring practice. Ground Water Monitoring Review 10, no. 4: 134–143. 10.1111/j.1745-6592.1990.tb00030.x

[gwat70022-bib-0031] Paradis, D. , and R. Lefebvre . 2013. Single‐well interference slug tests to assess the vertical hydraulic conductivity of unconsolidated aquifers. Journal of Hydrology 478: 102–118. 10.1016/j.jhydrol.2012.11.047

[gwat70022-bib-0032] Peffer, J.R. 1991. Complex aquifer‐aquitard relationships at an Appalachian plateau site. Groundwater 29, no. 2: 209–217. 10.1111/j.1745-6584.1991.tb00512.x

[gwat70022-bib-0033] Petersen, T.A. 2005. Hydrogeologic Cross Sections, Plate 9, 1:100,000. Geologic Atlas of Wabasha County, Falteisek J (proj manager). Minnesota. Minnesota Department of Natural Resources, Division of Waters County Atlas C‐14, Part B.

[gwat70022-bib-0034] Reidel, S.P. , V.G. Johnson , and F.A. Spane . 2002. Natural gas storage in basalt aquifers of the Columbia Basin, Pacific Northwest USA: A guide to site characterization, Rep. PNNL‐13962, Pacific Northwest National Lab, Richland, Washington, USA. 10.2172/15020781.

[gwat70022-bib-0035] Renaut, R.W. , and E.H. Gierlowski‐Kordesch . 2010. Lakes. In Facies Models 4, ed. N.P. James , and R.W. Dalrymple , 541–575. St. John's: Geological Association of Canada.

[gwat70022-bib-0036] Runkel, A.C. 2010. Southeastern Minnesota Paleozoic Hydrostratigraphy: Fractures in aquifers, Aquitards, and Aquitardifers: American Institute of Professional Geologists, Minnesota Chapter, Meeting Abstract, December 7, 2010. https://aipgmn.org/meetinginfo.php?id=27&ts=1368160930 (accessed March, 2025).

[gwat70022-bib-0037] Runkel, A.C. , J.R. Meyer , R.G. Tipping , J.R. Steenberg , A.J. Retzler , and B.L. Parker . 2019. Understanding Bedrock Fracture Flow to Improve Groundwater Quality, Minnesota Geological Survey Open File Report 25‐01. https://hdl.handle.net/11299/272537 (accessed March, 2025).

[gwat70022-bib-0038] Runkel, A.C. , R.G. Tipping , J.R. Meyer , J.R. Steenberg , A.J. Retzler , B.L. Parker , J.A. Green , J.D. Barry , and P.M. Jones . 2018. A multidisciplinary‐based conceptual model of a fractured sedimentary bedrock aquitard: Improved prediction of aquitard integrity. Hydrogeology Journal 26, no. 7: 2133–2159. 10.1007/s10040-018-1794-2

[gwat70022-bib-0039] Runkel, A.C. , J.R. Steenberg , R.G. Tipping , S. Janzen , and A.J. Retzler . 2015. Hydraulic Conductivity and Hydrostratigraphy of the Platteville Formation, Twin Cities Metropolitan Area, 28. Minnesota: Minnesota Geological Survey OFR 15‐01. https://hdl.handle.net/11299/171967 (accessed March, 2025).

[gwat70022-bib-0040] Runkel, A.C. , R.G. Tipping , J.A. Green , P.M. Jones , J.R. Meyer , B.L. Parker , J.R. Steenberg . A.J. Retzler . 2014. Hydrogeologic properties of the St. Lawrence Aquitard, southeastern Minnesota. Minnesota Geological Survey Open File Report 14–04: 119 p. https://hdl.handle.net/11299/165299 (accessed March, 2025).

[gwat70022-bib-0041] Runkel, A.C. , R.G. Tipping , E.C. Alexander Jr. , J.A. Green , J.H. Mossler , and S.C. Alexander . 2003. Hydrogeology of the Paleozoic bedrock in southeastern Minnesota. Minnesota Geological Survey Report of Investigations 61: 105 p, 1 map. https://hdl.handle.net/11299/58813 (accessed March, 2025).

[gwat70022-bib-0042] Sharp, J.M. Jr. 2023. A glossary of hydrogeology. Guelph, Ontario: The Groundwater Project. 10.21083/978-1-77470-079-2

[gwat70022-bib-0043] Shepley, M.G. 2024. Vertical hydraulic conductivity and layered heterogeneity: From measurements to models. Hydrogeology Journal 32: 1017–1042. 10.1007/s10040-024-02773-3

[gwat70022-bib-0044] Skinner, C. 2019. High‐resolution hydrogeological characterization of a fractured dolostone municipal supply aquifer to create a refined 3‐D conceptual site model with hydrogeologic units. MSc thesis, University of Guelph, Guelph, ON, Canada. https://atrium.lib.uoguelph.ca/items/0c38d9a4‐c8cb‐4947‐a0e8‐2fb7d3b4f623 (accessed April, 2025).

[gwat70022-bib-0045] Swanson, S.K. , J.M. Bahr , K.R. Bradbury , and K.M. Anderson . 2006. Evidence for preferential flow through sandstone aquifers in Southern Wisconsin. Sedimentary Geology 184, no. 3–4: 331–342.

[gwat70022-bib-0046] Tipping, R.G. 2012. Characterizing groundwater flow in the Twin Cities metropolitan area, Minnesota a chemical and hydrostratigraphic approach. PhD dissertation, University of Minnesota, Minneapolis, Minnesota: 186 p plus appendices.

[gwat70022-bib-0047] Van der Gun, J. 2022. Large aquifer systems around the world. In The Groundwater Project. Guelph, ON: The Groundwater Project Guelph. 10.21083/978-1-77470-020-4

[gwat70022-bib-0048] Whiteman, K.J. , J.J. Vaccaro , J.B. Gonthier , and H.H. Bauer . 1994. The hydrogeologic framework and geochemistry of the Columbia Plateau Aquifer System, Washington, Oregon, and Idaho: U.S. Geological Survey Professional Paper 1413‐B: 73 p. 10.3133/pp1413B

[gwat70022-bib-0049] Woessner, W.W. , and E.P. Poeter . 2020. Hydrogeologic Properties of Earth Materials and Principles of Groundwater Flow. Guelph, ON: The Groundwater Project.

[gwat70022-bib-0050] Zeng, L.B. , L. Gong , Y.Z. Zhang , S.Q. Dong , and W.Y. Lyu . 2023. A review of the genesis, evolution, and prediction of natural fractures in deep tight sandstones of China. American Association of Petroleum Geologists Bulletin 107, no. 10: 1687–1721. 10.1306/07052322120

